# Transepithelial photorefractive keratectomy for myopia and myopic astigmatism

**DOI:** 10.22336/rjo.2025.63

**Published:** 2025

**Authors:** Paul Filip Curcă, Cătălina Ioana Tătaru, George Sima, Marian Burcea, Speranța Schmitzer, Călin Petru Tătaru

**Affiliations:** 1 Clinical Department of Ophthalmology, “Carol Davila” University of Medicine and Pharmacy, Bucharest, Romania; 2 Department of Ophthalmology, “Prof. Dr. Mircea Olteanu” Institute for Ophthalmological Emergencies, Bucharest, Romania; 3 Alcor Clinic Bucharest, Romania

**Keywords:** transepithelial photorefractive keratomileusis, Trans-PRK, myopia, astigmatism, E3 = European Eye Epidemiology, PRK = Photorefractive keratectomy, Trans-PRK = Transepithelial photorefractive keratectomy, FS-LASIK = Femtosecond laser assisted in situ keratomileusis, NSAID = non-steroidal anti-inflammatory, logMAR = Logarithm of the Minimum Angle of Resolution, CDVA = Corrected distance visual acuity, UCVA or UDVA = Uncorrected distance visual acuity, SPH D = spherical diopters, CYL D = cylinder diopters, µm = microns, s = second, HOA = higher order aberration, CH = corneal hysteresis, CRF = corneal resistance factor

## Abstract

**Aim:**

Myopia is a growing endemic, especially in the young age groups (E3: 47.2% in 25-29 years old), which is projected to affect up to a staggering 50% of the world’s population in the following decades. Transepithelial photorefractive keratomileusis is a novel single-laser refractive surgery that offers advantages in simplicity and corneal biomechanics.

**Methods:**

The study reported Trans-PRK results on 71 patients (a total of 137 eyes) with myopia and myopic astigmatism.

**Results:**

Mean uncorrected visual acuity at 6 months was 0.02054 logMAR (0.9689 decimal) and at 1 year, 0.02 logMAR (0.9683 decimal); both values were superior to preoperative corrected visual acuity (CDVA). No statistical difference was found between 1 or more months postoperative UCVA and preoperative CDVA (p=0.848-0.723). The efficacy index was over 90% at 1 month and onwards. The safety index was 97% at 1 week and closer to 100% at later follow-up. Cumulative VA 20/20 was achieved in 82.6-89.2% of patients, and 20/25 in 94.2-95.1% of patients. Spherical refraction was +0.13 from plano at 1 month and -0.13 at 6 months; -0.17 at 1 year. Spherical equivalent accuracy was within ±1 D in 87.2% to 95.1% of patients. Vectorial correction index (ideal 1) indicated slight initial overcorrection at 1.19 at 1 month, 1.1 at 6 months, and an almost ideal 1.01 at 1 year; difference vector measured 0.11-0.19D.

**Discussion:**

The study reported excellent results using Trans-PRK for the treatment of myopia, as measured by cumulative postoperative uncorrected distance visual acuity, with refractive results aligning with those of comparable studies. Vectorial astigmatism correction was excellent, especially at the 1-year postoperative follow-up, approaching the ideal value. Thus, Trans-PRK offers the expected high-quality correction while presenting biomechanical and simplicity advantages over comparable techniques.

**Conclusions:**

Trans-PRK achieved excellent refractive results, a safe profile, and nearly ideal vectorial correction at later follow-up.

## Introduction

Myopia and astigmatism are refractive errors that are highly prevalent in today’s world.

Myopia mainly affects younger individuals, with the European Eye Epidemiology (E3) Consortium observing a peak prevalence in the 25-29 age group (47.2%) [1]. Other studies have reported incredibly high prevalence rates of 80-90% in East and Southeast Asia [2,3]. The myopic population is ever-increasing [4,5], with Holden et al. [6] analysis suggesting that up to 5 billion people could be myopic by 2050 [6], accounting for 50% of the entire population [6] and 10% of the population being highly myopic [6]. Implicated in this change are lifestyle elements essential to today’s life, such as increasing education levels, near-sighted activity, electronic device use, and reduced light levels (increase in indoor lighting and decreased exposure to natural outdoor light) [2,4-6]. The E3 Consortium has reported the prevalence of astigmatism in European populations to be 15-25% across various age categories [4] and at 24% in the United States population of the 2000s [5], as noted by Valluru G et al. [7]. The authors also reported an increase in astigmatism from the 1970s to the 2000s [5]. Photorefractive keratectomy (PRK) has undergone significant evolution since its introduction in the 1980s as the first widely accepted corneal laser-assisted refractive surgery method [8]. The initial alcohol-assisted epithelial debridement was replaced with precise laser-based epithelial removal [9,10], achieving more rapid visual recovery, accelerated healing, and less postoperative pain [10]. The use of the same excimer laser for both epithelial and stromal ablations enables a single-step refractive surgery [9,11,12]; this technique is referred to as transepithelial keratectomy (Trans-PRK) [9,11,12]. Newer laser-application algorithms have sought to achieve a smoother surface post-epithelial debridement, such as Smart Pulse Technology [13]. Reitblat O et al. explored performing the procedure for myopia in single-visit surgery versus planned multi-visit surgery. They found similar safety for both [14], similar astigmatism correction, and only a slight gain in accuracy of spherical correction for the multi-visit group of 0.07 diopters (63% versus 69% within ±0.5D) [14]. Compared to femtosecond laser-assisted in situ keratomileusis (FS-LASIK), Zhang J reported slightly better clinical outcomes for Trans-PRK in correcting high myopia [15]. Other studies have reported similar results, irrespective of the technique chosen [16,17]. These advancements in Trans-PRK create the groundwork for a surgery that is both reduced in complexity and effective, while minimizing the postoperative stromal and epithelial recovery period.

## Methods

**Fig. 1:**
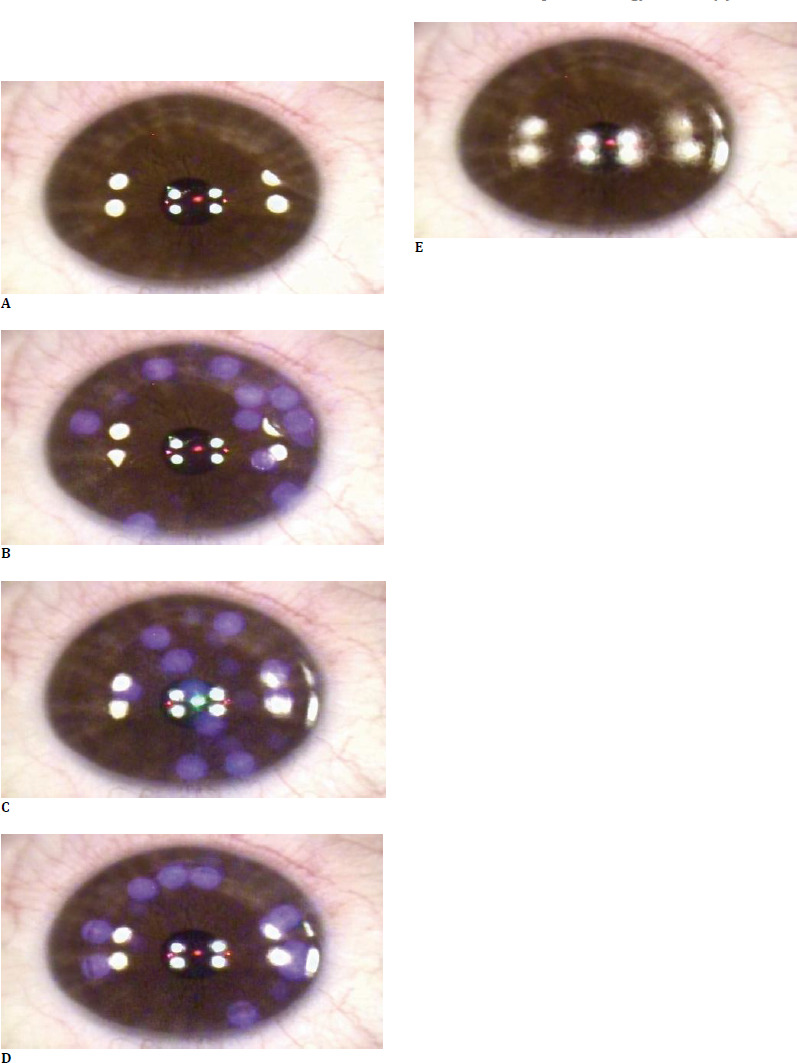
Refractive surgery from the study using the transepithelial keratectomy technique via the EX500 excimer laser. The surgery involves two steps: first, epithelial removal (**A-E**), and second, stromal ablation (**A-E**). The aspect before epithelial removal (**A**); the eye surface has been cooled using cold physiological serum and dried with a surgical sponge. The excimer laser progressively removes the epithelium (**B-D**). The stroma is left exposed for the next ablation step (**E**)

The study enrolled 71 patients (a total of 137 eyes) with myopia and myopic astigmatism who underwent Trans-PRK refractive surgery at the Alcor Clinic. Written informed consent was obtained before study participation, and the patient had the right to withdraw at any time during the study. The study was approved by the Ethics Committee of the Clinical Hospital for Ophthalmological Emergencies, Bucharest, Romania. Selection criteria for the study included: minimum age of 18 years, myopia or myopic astigmatism, stable refractive measurement for at least 1 year preoperatively, absence of corneal or lens-induced pathology, or previous laser-assisted refractive surgery. All surgeries were performed by the same experienced surgeon using the Alcon Wavelight EX500 ultraviolet excimer laser (Alcon, Forth Worth, Texas, United States) as part of the Wavelight Refractive Surgery Platform. Preoperative measurements were taken using the Wavelight Oculyzer II Scheimpflug camera and Topolyzer Vario corneal tomograph. Before laser ablation, cold physiological serum was irrigated on the corneal surface for cooling purposes. Afterwards, a surgical sponge was used to obtain a uniformly dry surface. Epithelial removal was performed using the EX500 laser (**Fig. 1**); depending on the set parameters, one or two breaks of approximately 10 seconds in laser treatment application were required to follow the thermal safety profile. After a brief 10-to 20-second pause, the stromal ablation step was performed uninterrupted, taking approximately 8 to 15 seconds, depending on the target parameters (**Fig. 1A-E, 2A-E**). The stromal bed was debrided using a surgical sponge with the application of Mitomycin-C 0.02%. Physiological serum was used to irrigate the final corneal surface. A therapeutic contact lens was applied in the operating room at the end of the surgery. The patients were instructed to follow topical antibiotic and anti-inflammatory treatments, in conjunction with an oral non-steroidal anti-inflammatory (NSAID). They were recommended to attend complete postoperative follow-up at 1 day, 1 week, 1 month, 6 months, and 1 year postoperatively. Data analysis and graphic data analysis were performed using an EXCEL database (Microsoft Corporation, Redmond, Washington, United States); statistical analysis was performed using Minitab 20 (Minitab Limited Liability Corporation, Coventry, United Kingdom) and the latest SPSS subscription version (International Business Machines Corporation, New York, United States). Visual acuity data were converted from decimal to Logarithm of the Minimum Angle of Resolution (logMAR) form for statistical analysis purposes and to Snellen for graphical representation. Vector analysis was performed using the AstigMATIC software as presented by Gauvin M and Wallerstein A [18].

**Fig. 2:**
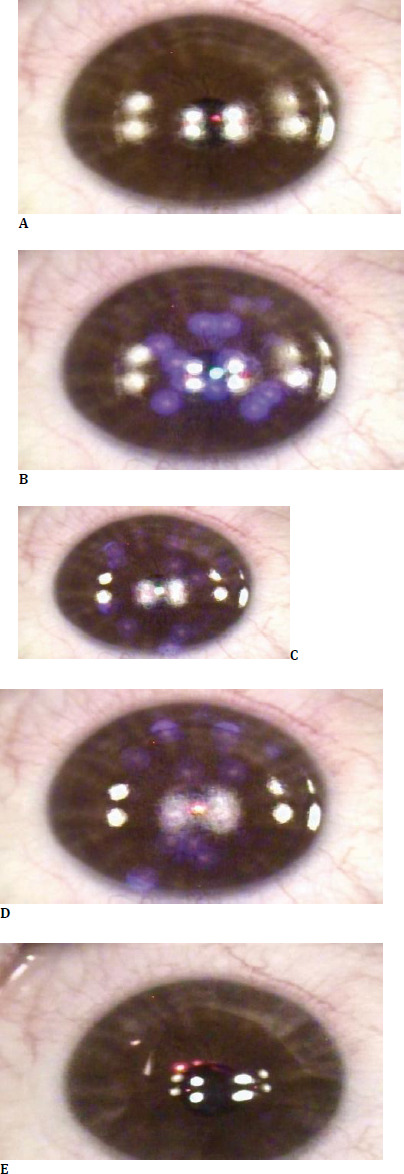
The stroma is left exposed for the next ablation step (**A**); Stromal ablation produces the desired corrective result (**B-D**). The debrided stroma is discarded using a mitomycin-C-impregnated sponge. Afterwards, the surface is washed using physiological serum. A bandage contact lens is applied at the end of the surgery (**E**)

## Results

### Visual acuity data

**Fig. 3:**
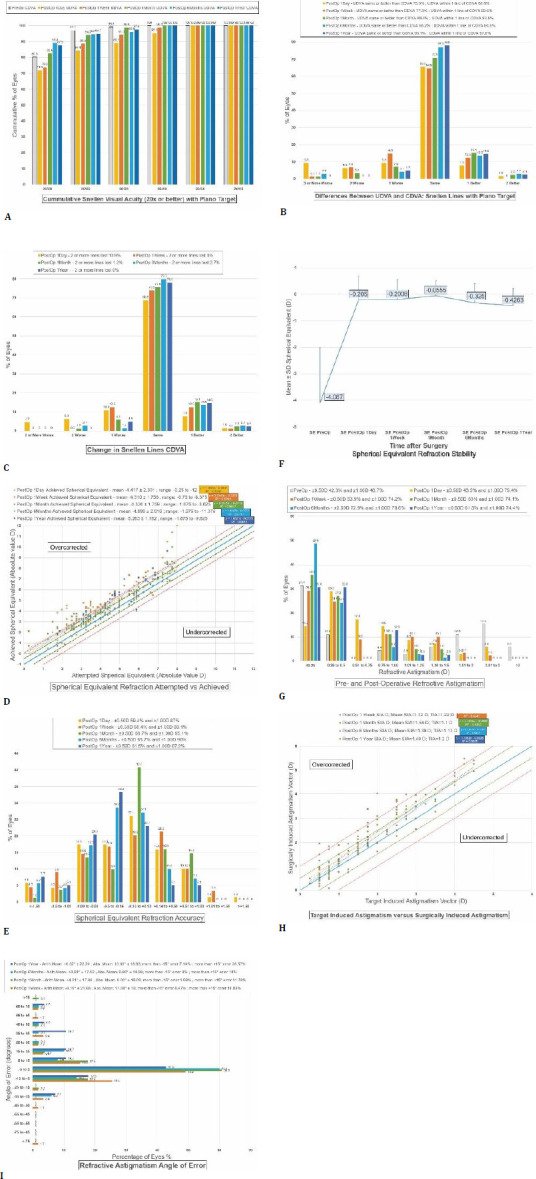
Nine standard refractive surgery graphs. The visual acuity results using Snellen lines (**A-C**); “Cumulative Snellen Visual Acuity (20x or better) with Plano Target” shows the percentage of patients with uncorrected vision at or above a specific Snellen line (**A**); “Differences Between UDVA and CDVA: Snellen lines with Plano Target” quantify the gain or loss of uncorrected distance visual acuity (UDVA) Snellen lines versus preoperative corrected distance visual acuity (CDVA) (**B**); “Change in Snellen Lines CDVA” quantifies the change between preoperative CDVA and postoperative CDVA (**C**); Refractive results expressed in spherical equivalent diopters (**D-F**). Spherical Equivalent (SE) Refraction Attempted versus (vs.) Achieved compares Attempted SE Correction (The laser target in Absolute SE) versus the achieved SE in absolute value. The deviation from the ideal diagonal lines represents over- or undercorrection (**D**); “Spherical Equivalent Refraction Accuracy” quantifies the accuracy of the achieved SE compared to a perfect plano target (0) (**E**). “Spherical Equivalent Refraction Stability” shows the stability of the achieved SE (**F**). Astigmatism refractive results (**G-I**). “Pre and Post-Operative Refractive Astigmatism” compares preoperative cylinder diopter values to postoperative cylinder diopter values (**G**); Vectorial calculations (**H, I**). “Target Induced Astigmatism (TIA) versus Surgically Induced Astigmatism (SIA)” compares vectorial results of TIA and SIA (**H**); “Refractive Astigmatism Angle of Error” quantifies the angle formed by the vectors of achieved correction (SIA) and intended correction (TIA); the results are presented in percentage form with standardized cutoff values (**I**)

For the 71 patients (137 eyes) in the study population, the mean corrected visual acuity (CDVA) was 0.0238 logMAR (0.96715 decimal). **Table 1** presents the detailed visual acuity data. Mean postoperative uncorrected visual acuity (UCVA) measured 0.0603 logMAR (0.9109 decimal) at 1 day, 0.04545 logMAR (0.9295 decimal) at 1 week, and 0.02535 logMAR (0.96512 decimal) at 1 month. Mean UCVA measured higher than preoperative CDVA at 6 months and 1 year postoperative: 0.02054 logMAR (0.9689 decimal) at 6 months; 0.02 logMAR (0.9683 decimal) at 1 year postoperatively. Statistical difference was recorded only between preoperative CDVA and 1-day and 1-week lower postoperative UCVAs (p=0.018 and 0.044 <0.05); no statistical difference was found between preoperative CDVA and postoperative UCVA at 1 month (p=0.848), 6 months (p=0.719), or 1 year (p=0.723). The gain or loss of Snellen lines is explored in detail in **Fig. 3B, C**. UCVA was the same or better as preoperative CDVA in 75% of patients at 1 day postoperatively, in 77.3% at 1 week, in 88.4% at 1 month, in 93.2% at 6 months and in 95.1% of patients at 1 year postoperative (**Fig. 3B**). Surgical efficacy or the efficacy index [19] was defined as an 0.8 or 80% percentage value cutoff of patients having UCVA measure at least or over preoperative CDVA (postoperative UCVA divided by preoperative CDVA) [19]. Surgical efficacy was achieved at 1 month postoperatively and exceeded 90% at 6 months and 1 year postoperatively. The Surgical Safety Index is defined as the percentage of patients who have not lost two or more lines of Snellen UCVA and have a cited cutoff level of 0.85 (85%) [19]. The safety index was calculated using cumulative Snellen lines, with values of 89.1% at 1 day postoperative, 100% at 1 week postoperative, 98.8% at 1 month, 97.3% at 6 months, and 100% at 1 year postoperatively. In total, 82.8% of patients were within 1 Snellen line of preoperative CDVA, 92.0% at 1 week, 93% at 1 month, 94.6% at 6 months, and 97.6% at 1 year (**Fig. 3B**). The highest percentage of 2 or more lines lost patients was at 1 day postoperatively at 10.9%; lowest at 1 week and 1 year postoperatively at 0%; 1.2% and 2.7% at 1 and 6 months postoperatively (**Fig. 3C**).

**Table 1:** Visual acuity data. The refractive surgery results are defined by efficacy (the surgery enabling vision without glasses or contact lenses) and by safety (if the patient does not receive the expected outcome, can they still use glasses/contact lenses and see as they did before the surgery?). CDVA = corrected distance visual acuity; UCVA = uncorrected distance visual acuity; Snellen lines = lines on the standardized visual acuity chart; gain refers to an increase in eyesight by seeing a smaller row of letters, while loss refers to a decrease in eyesight requiring increased size of the letters

	Mean	StDev	Min	Max	Variance	Coef. Of Var.
Patient Age (years)	24.747	±7.662	18	45	38.670	25.47
**Efficacy: Preop. CDVA versus Postop. UCVA**						
Preoperative CDVA logMAR	**0.0238**	0.05339	0	0.3	0.00285	224.36
1 Day UCVA logMAR	0.0603	0.1149	0	0.4	0.0132	190.58
1 Week UCVA logMAR	0.04545	0.09035	0	0.4	0.00816	198.77
1 Month UCVA logMAR	0.02535	0.06215	0	0.3	0.00386	245.19
6 Months UCVA logMAR	**0.02054**	0.6695	0	0.3	0.00448	325.93
Postop. 1 Year UCVA logMAR	**0.02**	0.06164	0	0.3	0.00380	308.22
**Efficacy: Snellen Lines Change Preop CDVA versus Postop UCVA**						
Postop. 1 Day Change	**+0.1719**	0.4199	0	+2	0.1763	244.32
Postop. 1 Week Change	**+0.2159**	0.4661	0	+2	0.2172	215.86
Postop. 1 Month Change	**+0.0930**	0.3300	0	+2	0.1089	354.74
Postop. 6 Months Change	**+0.0541**	0.2277	0	+1	0.0518	421.19
Postop. 1 Year Change	**+0.0732**	0.2637	0	+1	0.0695	360.32
**Safety: Preop. CDVA versus Postop. UCVA**						
Preoperative CDVA logMAR	0.0238	0.05339	0.00	0.3	0.00285	224.36
1 Day CDVA logMAR	0.0431	0.0869	0	0.3	0.0076	201.60
1 Week CDVA logMAR	0.02318	0.05566	0	0.3	0.00310	240.09
1 Month CDVA logMAR	0.00435	0.04031	0	0.3	0.00163	262.65
6 Months CDVA logMAR	0.00662	0.05698	0	0.3	0.00325	363.50
1 Year CDVA logMAR	0.01707	0.05433	0	0.3	0.00295	318.19
**Safety: Snellen Lines Change Preop CDVA versus Postop CDVA**						
Postop. 1 Day LChange	-0.266	0.930	-3	+2	0.865	-350.1
Postop. 1 Week Change	+0.0227	0.5462	-1	+2	0.2983	2403.25
Postop. 1 Month Change	+0.1163	0.5825	-2	+2	0.3393	500.92
Postop. 6 Months Change	+0.1216	0.5957	-2	+2	0.5957	489.80
Postop. 1 Year Change	+0.1463	0.5273	-1	+2	0.5273	360.22

**Fig. 4:**
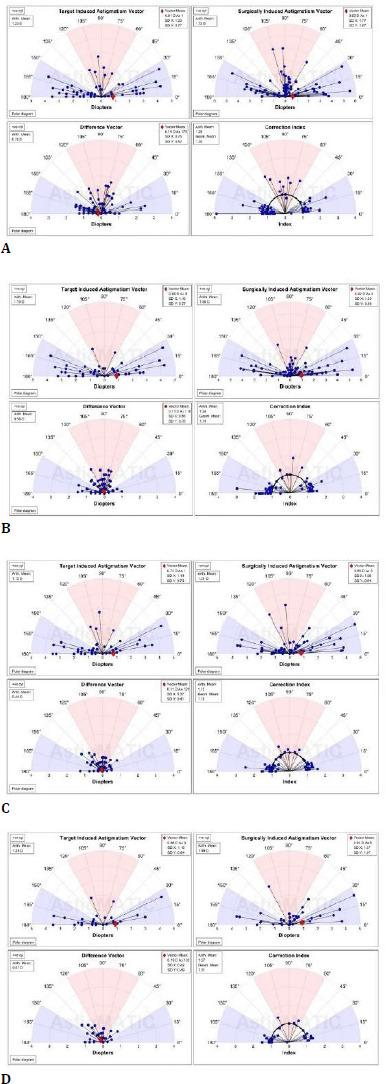
Results of data analysis using the AstigMATIC software for vectorial calculations [18], 1 week postoperatively (**A**), 1 month postoperatively (**B**), 6 months postoperatively (**C**), 12 months postoperatively (**D**). The terms represent astigmatism terminology as discussed in the Alpins method [21]. Target-induced astigmatism (TIA) quantifies the intended astigmatism treatment at the corneal plane [21] and represents the astigmatic change that the surgery was designed to cause [21]. Surgically Induced Astigmatism (SIA) is the actual measured change in astigmatism that the surgery produced [21]. The correction index is the ratio of SIA divided by TIA [21]. The correction index has an ideal value of 1; overcorrection results in values greater than 1, while undercorrection results in values less than 1 [21]. The Difference Vector reflects the adjustment to the surgical result that would have enabled the initial surgery to achieve its intended target [21]

### Refractive data

Refractive results are presented in-depth in **Table 2**. Preoperatively, the mean refraction was -3.706 spherical diopters (SPH D), the mean cycloplegic refraction -3.373 SPH D, and the mean manifest refraction (used for CDVA correction) -3.529 SPH D. Postoperatively, refraction measured +0.127 SPH D at 1 day, +0.0758 SPH D at 1 week, +0.1358 SPH D at 1 month, -0.1357 SPH D at 6 months, and -0.1731 SPH D at 1 year. A slight trend of initial overcorrection with values above 0 SPH D was noted, followed by slight undercorrection at 6 months and 1 year postoperatively. Preoperative astigmatism measured -1.1796-cylinder diopters (CYL D), -1.2183 CYL D with cycloplegia, and -1.097 CYL D manifest for CDVA correction. Postoperatively, residual astigmatism measured -0.663 CYL D at 1 day, -0.5534 CYL D at 1 week, -0.3826 CYL D at 1 month, -0.3786 CYL D at 6 months, and -0.5064 CYL D at 1 year postoperatively. Postoperative refractive astigmatism was within ±0.5 D of the plano target in 42.3% of patients preoperatively, 43.5% at 1 day postoperatively, 53.9% at 1 week, 63% at 1 month, 72.9% at 6 months, and 61.5% at 1 year (**Fig. 3G**). Postoperative refractive astigmatism was within ±1.00D in 46.7% of patients preoperatively and in 75.4% at 1 day postoperatively, 74.2% at 1 week, 74.1% at 1 month, 78.6% at 6 months, and 74.4% at 1 year (**Fig. 2G**). Residual astigmatism was greatest immediately after the surgery, stabilized at 1-6 months postoperatively, and slightly increased at the 1-year follow-up. Refractive astigmatism angle of error results were best at 1 and 6 months (60.8% and 60% respectively, within a 5-degree tolerance from ideal 0 (**Fig. 2I**). Mean preoperative spherical equivalent (SE) target was the mean of the set laser-correction target in spherical equivalent (spherical diopters + ½ cylinder diopters). Preoperative SE was -4.087 D. Postoperatively, SE was -0.205 at 1 day, -0.2008 at 1 week, -0.0555 at 1 month, -0.325 at 6 months, and -0.4263 at 1 year postoperative. Postoperative SE was within ±0.5 D of plano refraction in 59.4% of patients at 1 day, 58.4% at 1 week, 66.7% at 1 month, 65.7% at 6 months, and 61.5% at 1 year (**Fig. 2E**). Postoperative SE was within ±1.00 D in 87% of patients at 1 day, 83.1% at 1 week, 95.1% at 1 month, 90% at 6 months and 87.2% at 1 year (**Fig. 2E**). Considering previous spherical refraction data, the resultant trend was towards initial overcorrection, followed by stability at 1-6 months spherical and 1-month SE. Subsequently, the values drifted slightly into undercorrection at 6 months and 1 year preoperatively.

**Table 2:** Refractive results. This table compares pre- and postoperative data about spherical refraction in diopters (SPH D), cylinder refraction in diopters (CYL D), and spherical equivalent data (spherical + ½ cylinder diopters; SE or Spherical Equiv.)

	Mean	StDev	Min	Max	Variance	Coef. Of Var.
**Pre-Postop Refraction SPHD**						
Preoperative Refraction	-3.706	2.059	-11	0	4.241	-55.58
Preop. Cycloplegia Refraction	-3.373	2.107	-9.25	0	4.441	-62.48
Preop. Manifest Refraction	-3.529	3.878	-8.25	0	3.878	-55.80
1 Day Refraction	0.127	0.846	-3.25	+1.75	0.716	667.37
1 Week Refraction	0.0758	0.6830	-1.75	+1.75	0.4665	900.54
1 Month Refraction	0.1358	0.5047	-1.75	+1.5	0.2548	371.57
6 Months Refraction	-0.1357	0.6859	-3.25	+1.25	0.4704	-505.39
1 Year Refraction	-0.1731	0.5797	-1.5	+1.25	0.3360	-334.93
**Pre-Postop Refraction CYLD**						
Preoperative Refraction	-1.1796	1.1279	-4.75	0	1.2721	-95.61
Preop. Cycloplegia Refraction	-1.2183	1.1431	-4.75	0	1.3068	-93.83
Preop. Manifest Refraction	-1.097	1.160	-4.5	0	1.345	-105.70
1 Day Refraction	-0.663	0.8279	-3	+2.5	0.6853	-124.86
1 Week Refraction	-0.5534	0.7428	-2.5	+1.5	0.5518	-134.24
1 Month Refraction	-0.3826	0.5706	-1.5	+1.26	0.3255	-149.13
6 Months Refraction	-0.3786	0.4099	-1.5	+0.75	0.1680	-108.27
1 Year Refraction	-0.5064	0.4234	-1.5	+0.75	0.1792	-83.60
**Pre-Postop Sph. Equivalent D**						
Preop. SE Target	-4.087	2.076	-8	-1.5	4.311	-50.81
1 Day Spherical Equiv.	-0.205	0.905	-4	+2.125	0.818	-441.94
1 Week Spherical Equiv.	-0.2008	0.7509	-2.375	+1.25	0.5638	-373.85
1 Month Spherical Equiv.	-0.0555	0.5573	-2	+1	0.3106	-1004.33
6 Months Spherical Equiv.	-0.3250	0.7262	-3.625	+1	0.5274	-223.44
Year Spherical Equiv.	-0.4263	0.6215	-2.125	+0.75	0.3863	-145.80

### Ablation parameters and intraoperative data

The mean targeted correction was -3.513 ± 2.152 SPH D, -1.1478 ± 1.1507 CYL D, and in SE -4.087 ± 2.076. Average preoperative central corneal pachymetry was 545.68 ± 27.03 microns (µm).

Calculations for Trans-PRK indicated a required ablation of a mean 51.022 ± 8.427 µm of corneal tissue, resulting in a predicted residual stromal thickness of 427.13 ± 36.43 µm. On the Alcon Wavelight refractive surgery platform, the MAX parameter refers to the necessary corneal ablation to correct higher-order aberrations [20]. In contrast, the CEN parameter is the amount of central corneal tissue removal [20]. MAX presented a mean of 68.30 ± 21.41 µm, while CEN presented a mean of 67.74 ± 22.47 µm.

The maximum predicted tissue ablation was 120 µm, and the highest values of MAX and CEN both equaled 112.96 µm. The lowest MAX was 22.96 µm, while the lowest CEN was 0 µm. Depending on the set parameters, the ablation and optical zones could be larger or more restricted in cases of high diopter correction. On average, the whole ablation zone measured 8.3635 ± 0.8833 µm, the optical zone 6.3781 ± 0.5534 µm, and the transition zone 1.015 ± 0.6399 µm. Concerning the overall treatment duration (including breaks). Trans-PRK required a minimum of 10 seconds, an average of 47.715 ± 10.18 s, and a maximum of 77 s. This treatment duration required short breaks for thermal and safety purposes, with an average of 1.4672 ± 0.748 breaks required, ranging from a minimum of 0 to a maximum of 4. In total, the breaks cumulated a mean of 14.949 ± 5.719 s and a maximum of 42 s.

### Vectorial analysis

Vector analysis was performed using the AstigMATIC software as presented by Gauvin M and Wallerstein A [18], with the results detailed in **Table 3, Fig. 3**. Target-induced astigmatism (TIA) quantifies the intended astigmatism treatment at the corneal plane [21] and is the astigmatic change the surgery was designed to induce [21]. Surgically Induced Astigmatism (SIA) is the actual measured change in astigmatism that the surgery produced [21]. The correction index is the ratio of SIA divided by TIA [21]. The correction index has an ideal value of 1; overcorrection results in values greater than 1, while undercorrection results in values less than 1 [21]. Angle of error is the angle formed by the vectors of achieved correction (SIA) and intended correction (TIA). The Difference Vector reflects the adjustment to the surgical result that would have enabled the initial surgery to achieve its intended target [21]. In the study, the Difference Vector was lowest at 1 and 6 months postoperatively (0.11D) and had a similar value at 1 week and 1 year (0.19D); however, the Correction Index reflecting under/overcorrection was closer to the ideal one at 1 year postoperatively (1.01 geometric mean and 1.07 arithmetic mean) and second-closer to 1 at 6 months postoperatively (1.1 geometric mean and 1.12 arithmetic mean). As such, it appears that the surgical results begin to stabilize around 1 month postoperatively (Difference Vector values). The values for the Difference Vector slightly shift at 1 year postoperatively; however, the overall Corrective Index remains excellent and is best at 6 months or more following the surgery.

**Table 3:** Vectorial astigmatism results as calculated using the AstigMATIC software (Gauvin M and Wallerstein A [18]). Target-Induced Astigmatism (TIA) refers to the intended change in the corneal plane. Surgically Induced Astigmatism (SIA) is the actual change induced by the treatment at the corneal plane. The corrective index is the ratio of SIA divided by TIA and indicates undercorrection <1, ideal result 1, or overcorrection >1. The residual astigmatism after surgery is described by the geometric mean of the Difference Vector; thus, it represents the adjustment to the original surgery that would have ideally reached the target

Postop. time period	Target Induced		Surgically Induced		Difference Vector		Correction Index	
	Astigmatism		Astigmatism					
	Vector (TIA)		Vector (SIA)					
	Vector	Arithmetic	Vector	Arithmetic	Vector	Arithmetic	Vector	Arithmetic
	Mean	Mean	Mean	Mean	Mean	Mean	Mean	Mean
1 week	0.81D Ax 1°	1.22D	0.63D Ax 4°	1.72D	0.19D Ax 172°	0.74D	1.20	1.29
1 month	0.85D Ax 2°	1.1D	0.92D Ax 4°	1.56D	0.11D Ax 118°	0.58D	1.19	1.24
6 months	0.74D Ax 1°	1.13D	0.81D Ax 5°	1.39D	0.11D Ax 121°	0.44D	1.1	1.12
1 year	0.86D Ax 3°	1.2D	0.91D Ax 9°	1.49D	0.19D Ax 132°	0.57D	1.01	1.07

**Table 3** presents the results of data analysis using the AstigMATIC software for vectorial calculations [18]. The terms represent astigmatism terminology as discussed in the Alpins method [21]. Target-induced astigmatism (TIA) quantifies the intended astigmatism treatment at the corneal plane [21] and represents the astigmatic change that the surgery was designed to cause [21]. Surgically Induced Astigmatism (SIA) is the actual measured change in astigmatism that the surgery produced [21]. The correction index is the ratio of SIA divided by TIA [21]. The correction index has an ideal value of 1; overcorrection results in values greater than 1, while undercorrection results in values less than 1 [21]. The Difference Vector reflects the adjustment to the surgical result that would have enabled the initial surgery to achieve its intended target [21].

## Discussion

Myopia and myopic astigmatism are highly prevalent refractive errors [1-6], and according to recent studies, this prevalence is set to grow explosively as societal and lifestyle factors clash with the eye optical system [5,6], possibly affecting nearly 6 billion people by 2050 (Holden et al.) [6]. In light of this, the burden placed on refractive surgery is only set to increase. Refractive surgery has undergone significant development, achieving both safety and efficacy through various surgical techniques [17]. Chang JY et al. conducted a comparative analysis of visual outcomes from previous studies [17], highlighting the favorable or equivalent efficacy and safety of Trans-PRK in myopic and high myopic patients compared to the LASIK technique, as well as a slight improvement in predictability of refraction, including results within ±0.5 D. Zheng Z et al. study [17,22] reported better UDVA, CDVA, and SE results at 1 month postoperatively with SMILE; the difference receded at 3 months after surgery [17,22]. Furthermore, vectorial parameters did not differ at 1-3 months postoperatively [22]. Thus, if a patient desires refractive surgery and is suitable for any of the refractive surgery techniques, such as LASIK, Trans-PRK, or SMILE, then either procedure would produce a safe and effective result for that patient. However, Trans-PRK could offer specific improvements. Trans-PRK has surface-shaping abilities and has been reported to produce lower amounts of higher-order aberration (HOA) and coma [22] (versus SMILE) in refractive predictability [17] (versus LASIK). Concerning the cornea itself, Kamiya et al. studied the effect of PRK and LASIK refractive surgery by using a rapid air impulse to produce applanation of the cornea. They monitored the result using an electron optical system [23]. Both surgeries altered corneal hysteresis and the corneal resistance factor depending on the amount of myopic correction [23]. The author reported that LASIK significantly decreased CH and CRF more than PRK, and theorized that PRK, the technique preceding the advent of Trans-PRK, might be a biomechanically less invasive approach than LASIK. Guo H et al. conducted a meta-analysis of 5 studies, which also found a clear advantage for PRK and SMILE over FS-LASIK in corneal biomechanics [23]. To our knowledge, data of CH and CRF with Trans-PRK has not yet been reported [17].

Our study reported overall excellent results using the Trans-PRK surgical technique for the treatment of myopia. UCVA measured the same or better for over 90% of patients at 6 months and 1 year postoperatively. In total, 82.8% of patients were within 1 Snellen line of preoperative CDVA, 92.0% at 1 week, 93% at 1 month, 94.6% at 6 months, and 97.6% at 12 months. The safety index was excellent at 97% at 1 week postoperatively. The percentage of patients with two or more lines lost was 10.9% at 1 day postoperatively. The lowest rates were 0%, 1.2%, and 2.7% at 1 week, 1 year, and 6 months, respectively. The visual acuity results were favorable when compared to the large-population study of Reitblat O et al. [14] with our study reporting cumulated visual acuity of 20/20 in 82.6% at 1 month, 89.2% at 6 months and 87.8% at 1 year (versus 42.1% and 44.9 % in the comparison study [14]); for 20/32 cumulated VA the difference was 95.9-98.8% in this study versus 91-92.9% in the comparative study. Comparison with other studies was more in line with Aslanides IM et al. [12] study, which reported 20/25 cumulative VA of 97.1% for Trans-PRK, higher than the present study, 94.2%-94.6%- 95.1% (1-6-12 months postoperatively), and lower 20/20 VA in 77.1% versus our study’s 82.6-89.2%.

Zhang J et al. reported the highest VA of 20/20 in 98% of patients [15], and Zheng Z et al. reported the second-highest of 93% of patients [22]. Our study, however, reported lower refractive accuracy for within ±0.5 D and comparative accuracy for % within ±1 D. Postoperative SE was within ±0.5 D of plano refraction in 59.4% of patients at 1 day, 58.4% at 1 week, 66.7% at 1 month, 65.7% at 6 months and 61.5% at 1 year and for within ±1.00 D in 87% of patients at 1 day, 83.1% at 1 week, 95.1% at 1 month, 90% at 6 months and 87.2% at 1 year. Aslanides IM et al. [12] reported higher 91.4% within ±0.5 D and 97.1% within ±1 D. Reitblat O et al. [14] reported similar results to our study at 63.3% or 69.0% within ±0.5 D and 89.5% or 90.2% within ±1 D. Zhang Z reported 94.5% of eyes within ±0.5 D [22] and Zhang J 87% within ±0.5 D and 100% within ±1D [15]. The Difference Vector reflects the error from an ideal surgical result [21] and was lowest at 1 and 6 months postoperatively, with an excellent value of 0.11D, and was similar at 1 week and 1 year (0.19D). The Correction Index (SIA/TIA ratio) was very close to the ideal one value at 1 year postoperatively (1.01 geometric mean and 1.07 arithmetic mean) and second-closest to 1 at 6 months postoperatively (1.1 geometric mean and 1.12 arithmetic mean). Thus, the surgical results began to stabilize approximately 1 month postoperatively (as indicated by the Difference Vector values); the values for the Difference Vector shifted slightly at 1 year postoperatively. Overall, the corrective power of the surgery was very good at 6 months or more after the surgery. It had an almost ideal Corrective Index of 1.01 at 1-year follow-up.

Transepithelial photorefractive keratectomy proved a reliably effective and safe technique, achieving good results in the present study. Refractive surgery, particularly for myopia, has advanced to the point that only minor differences separate the methods of SMILE, LASIK, and Trans-PRK [17]. All techniques meet the criteria for effectiveness and safety [17].

Considering this, Trans-PRK could reduce the complexity of the intervention and be friendlier with corneal biomechanical stability versus LASIK [23,24], offer more personalization of the surface ablation versus SMILE, and have fewer HOAs [22]. Despite these supported advantages, PRK remains a non-inferior, safe, and practical option for refractive surgery. Further improvement upon the technique will only offer benefit to the patient [13,17].

## Conclusions

Transepithelial photorefractive keratectomy is a technological evolution of the PRK surgical technique, achieving effective and safe results in the present study. In the ever-evolving field of refractive surgery, Trans-PRK offers a simplified, single-laser solution for correcting myopia.
